# Enhancement of Mitochondrial Homeostasis: A Novel Approach to Attenuate Hypoxic Myocardial Injury

**DOI:** 10.7150/ijms.103986

**Published:** 2024-10-28

**Authors:** Zhijiang Guo, Yingjie Tian, Jing Gao, Bei Zhou, XiuTeng Zhou, Xing Chang, Hao Zhou

**Affiliations:** 1 Guang'anmen Hospital, China Academy of Chinese Medical Sciences, Beijing, 100053, China.; 2 Beijing University of Chinese Medicine, Beijing, 100028, China.; 3 Center for Drug Evaluation, National Medical Products Administration, Beijing, China.; 4 National Resource Center for Chinese Materia Medica, China Academy of Chinese Medical Sciences/State Key Laboratory for Quality Assurance and Sustainable Use of Dao-di Herbs, Beijing, 100700, China.; 5 Senior Department of Cardiology, The Sixth Medical Center of People's Liberation Army General Hospital, Beijing, China.

**Keywords:** hypoxic myocardial injury, mitophagy, unfolded protein response (UPR)

## Abstract

Cardiomyocytes are highly oxygen-dependent cells, relying on oxygen-driven oxidative phosphorylation to maintain their function. During hypoxia, mitochondrial ATP production decreases, leading to calcium overload, acidosis, and oxidative stress, which collectively trigger myocardial injury. Ischemic heart disease, caused by coronary atherosclerosis, results in myocardial ischemia and hypoxia, leading to ischemia-reperfusion (I/R) injury. Early myocardial injury is attributed to ischemia and hypoxia, but even after thrombolytic therapy, interventional surgery, or coronary artery bypass grafting (CABG) restores local blood flow and oxygen supply, myocardial reperfusion injury (I/R) may still occur. Mitochondria, often referred to as the "powerhouses" of the cell, play a crucial role in cellular energy production. In the early stages of ischemia and hypoxia, mitochondrial dysfunction disrupts mitochondrial homeostasis, causing the accumulation of unfolded or misfolded proteins in the mitochondria. This activates the mitochondrial unfolded protein response (mtUPR) and mitophagy, which work to clear damaged proteins and mitochondria, playing a key role during this period. This review focuses on mitochondrial mechanisms during the ischemic phase of ischemia-reperfusion injury, aiming to provide new theoretical foundations and potential therapeutic strategies to reduce myocardial damage.

## 1. Introduction

Ischemic heart disease is primarily caused by coronary atherosclerosis, leading to myocardial ischemia and oxygen deprivation. Within the spectrum of cardiovascular diseases, its incidence and mortality rates are notably high^1^. Thrombolytic therapy, percutaneous coronary intervention (PCI), and coronary artery bypass grafting (CABG) are currently the most direct and effective treatments for ischemic heart disease, as they restore blood supply to ischemic regions. Nevertheless, before blood flow is restored, hypoxia exacerbates myocardial damage and further expands the infarct area.

Mitochondria, long recognized as the cellular “powerhouses”, generate energy for cellular metabolism via oxidative phosphorylation (OXPHOS). Mitochondria consist of two membranes: the outer membrane (OMM) and the inner membrane (IMM). The IMM is subdivided into the inner boundary membrane (IBM), which connects to both the OMM and the cristae. Cristae connect to the IBM through narrow junctions and extend into the mitochondrial matrix, increasing the surface area of the IMM. The outer membrane faces the cytoplasm, while the inner membrane extends into the mitochondrial matrix, which contains mitochondrial DNA (mtDNA). The compartment between the inner and outer membranes is known as the intermembrane space (IMS). Mitochondria are responsible for ATP synthesis through the tricarboxylic acid cycle and oxidative phosphorylation, playing essential roles in nearly all eukaryotic cells^2^. In addition to energy production, mitochondria perform various other critical functions, such as protein folding, and communicate with the nucleus and other organelles to maintain cellular homeostasis. Maintaining mitochondrial homeostasis is critical for preventing hypoxia-induced myocardial injury^3^. Mitochondrial autophagy (mitophagy) and the mitochondrial unfolded protein response (UPRmt) are key mechanisms for preserving mitochondrial stability by clearing damaged mitochondria and misfolded or unfolded proteins. These mechanisms also help mitigate hypoxia-mediated myocardial damage, and targeting the UPRmt and autophagy pathways may offer new therapeutic strategies for treating hypoxia-induced myocardial injury^4^.

## 2. Myocardial hypoxic injury

**Figure 1:**
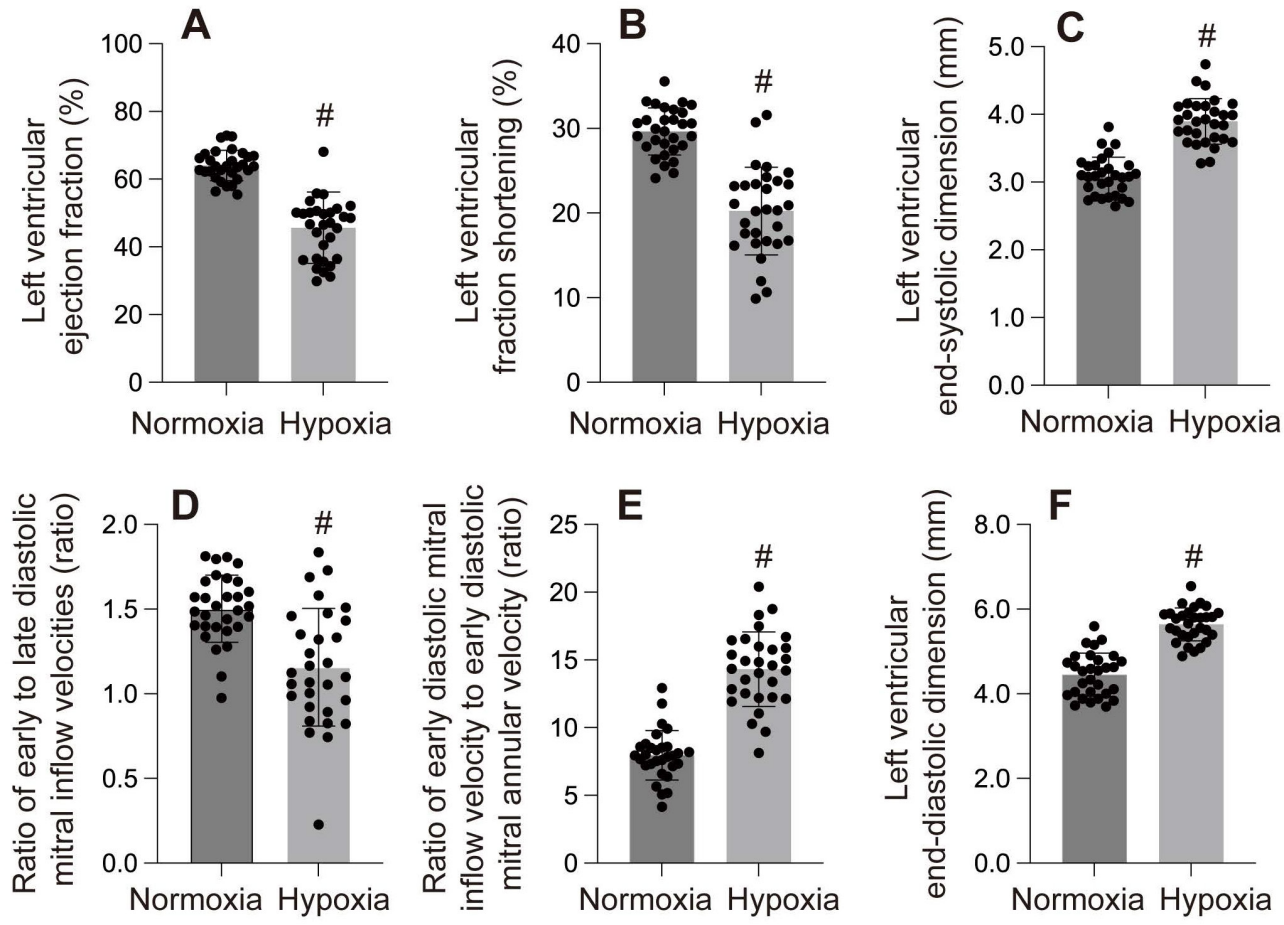
A-F: These panels display various left ventricular (LV) functional parameters under normoxia and hypoxia. #p < 0.05.

**Figure 2:**
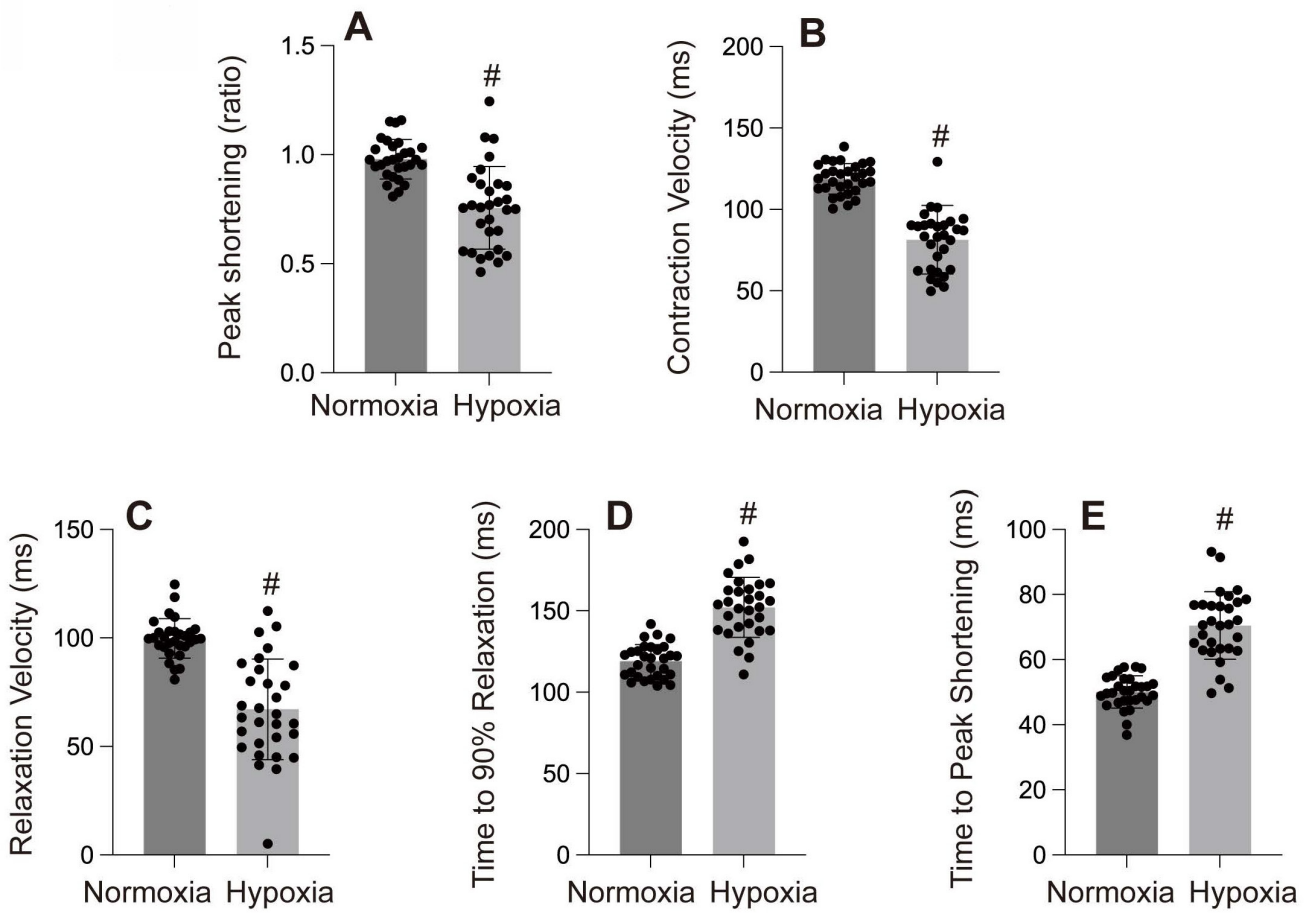
A-E: Cardiac contractility and relaxation indices. #p < 0.05.

**Figure 3:**
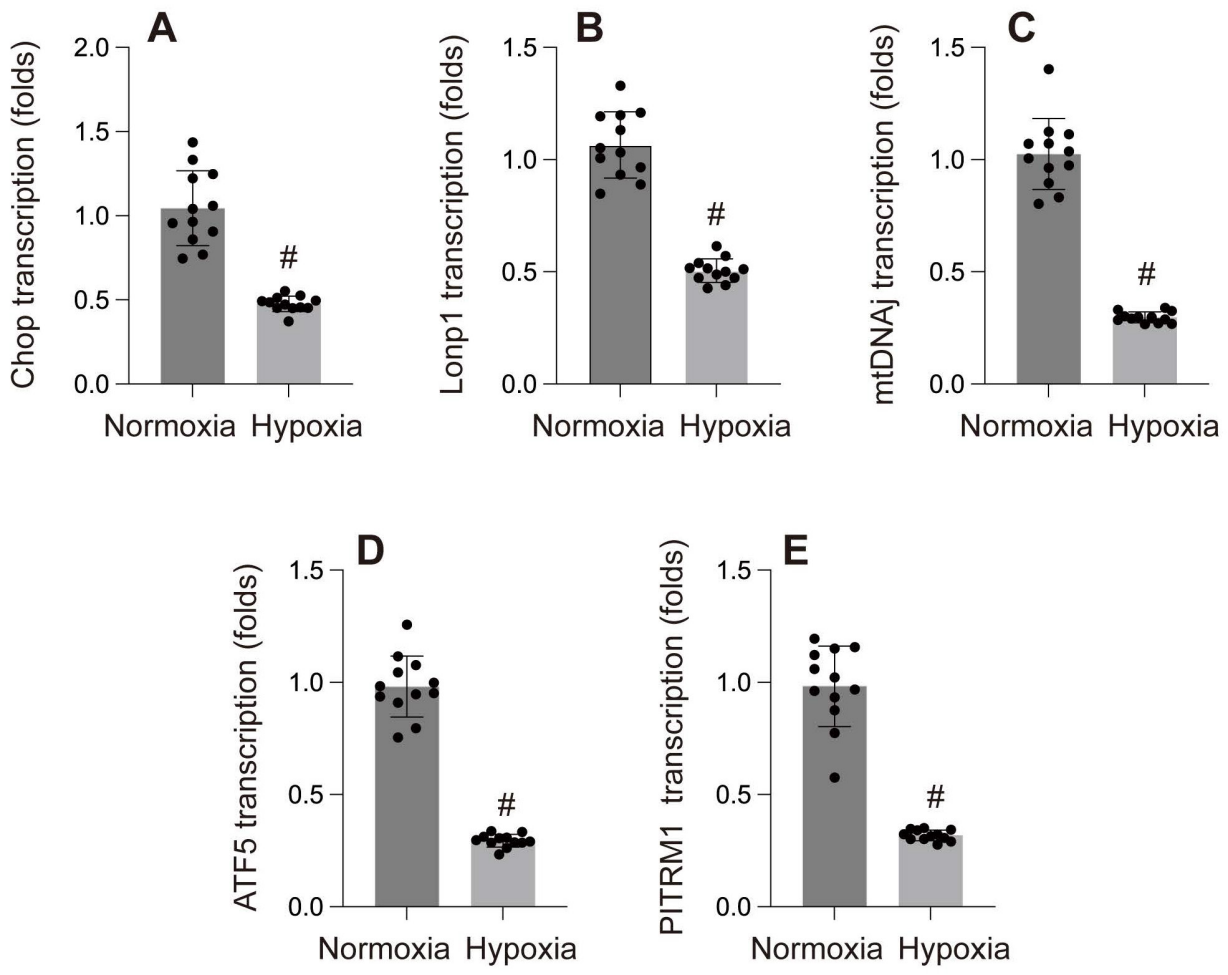
A-E: Gene transcription under normoxia and hypoxia. #p < 0.05.

Myocardial hypoxic injury can manifest as myocardial stunning, ventricular arrhythmias induced by hypoxic, vascular hypoxic injury, or fatal Myocardial hypoxic injury^5^ (Figure 1-3).

### 2.1. Myocardial stunning

Myocardial stunning refers to a reversible left ventricular dysfunction that represents a pathological response to myocardial cell injury triggered by ischemia.^6^ In the early stages of ischemic cardiomyopathy, myocardial injury arises from reduced oxygen supply to the myocardium^7^,^8^. At this stage, the myocardium experiences functional impairment and metabolic changes due to temporary hypoxia, typically without immediate structural changes. Functional impairment is primarily characterized by reduced myocardial cell contractility, leading to a decrease in left ventricular ejection fraction (LVEF), reduced myocardial output, and regional wall motion abnormalities. Metabolic changes include anaerobic glycolysis, which leads to lactic acid accumulation and reduced ATP production, causing harmful byproducts to accumulate and further impair normal myocardial cell function, thereby aggravating myocardial injury. At this early stage, the myocardium undergoes reversible injury, often observed as myocardial stunning, which is characterized by a temporary but reversible decrease in myocardial contractility without necrosis. Prolonged hypoxia ultimately results in myocardial hibernation, where myocardial cells downregulate their function to preserve viability during prolonged ischemia^9^. In this state, myocardial cells demonstrate reduced contractile function and blood flow. Although reperfusion can restore blood supply, the recovery of myocardial contractility is a gradual process.

### 2.2. Hypoxia-induced arrhythmias

Arrhythmia is the primary clinical manifestation of Myocardial hypoxic injury. After hypoxia, more than 80% of patients with acute myocardial infarction(AMI) will experience arrhythmias, such as ventricular tachycardia (VT) and ventricular fibrillation (VF)^11,12^. Patients with VT may experience palpitations, fatigue, dizziness, and chest discomfort^12^. Prolonged VT can decrease ventricular filling and pump efficiency, and in severe cases, it can cause decreased blood pressure and heart failure, posing a life-threatening risk to the patient^13^,^14^. Additionally, sustained VT can sometimes progress to VF. VF is a rapid and disorganized ineffective heartbeat that leads to cessation of blood supply to the body. Fainting and loss of consciousness are common symptoms of VF. It is the primary cause of sudden cardiac death, and without prompt treatment, it can rapidly result in death^15^. The existence of malignant hypoxic-induced arrhythmias in humans has been a topic of debate^16^. hypoxic-induced arrhythmia and myocardial stunning can both be seen as manifestations of sublethal and reversible cellular injury. Studies have shown that antioxidants or calcium antagonists can help alleviate these manifestations of hypoxic injury^17^,^18^.

### 2.3. Vascular hypoxic injury

Approximately 40% of patients with AMI experience microvascular obstruction (MVO) following thrombolysis or PCI, which is a severe form of reperfusion injury^17^. Clinically, MVO is characterized by the absence of reflow in the coronary artery related to the infarct after PCI. Although reperfusion relieves blood flow obstruction in the larger arteries, it still hampers flow in the smaller arteries, resulting in significant impairment of resting blood flow within the vessels and ultimately insufficient blood supply to the myocardium^19^. The disparity between reestablishing blood supply in the outer walls and the microvasculature in patients with no reflow is a crucial factor contributing to treatment failure^20^.

The pathophysiology of MVO is complex and multifactorial, primarily involving distal microembolization following angioplasty or thrombolysis, the formation of new thrombi due to complement and leukocyte activation, internal vascular factors, endothelial leakage-induced interstitial edema leading to small vessel narrowing, and direct endothelial cell toxicity and reperfusion injury-induced microvascular spasm^20^,^21^ Microvascular dysfunction, often referred to as the "no-reflow" phenomenon, frequently indicates undesirable cardiovascular outcomes in clinical practice. Clinically, MVO may be asymptomatic or cause symptoms such as angina, hemodynamic instability, or conduction changes^22^,^23^. Its diagnosis necessitates a comprehensive evaluation through angiography, electrocardiography, nuclear scintigraphy, myocardial contrast echocardiography, or cardiovascular magnetic resonance^20^,^24^,^25^.

### 2.4. Lethal myocardial hypoxic injury

Lethal damage is primarily driven by severe myocardial ischemia and hypoxia, ultimately culminating in myocardial cell death. Prolonged hypoxia induces adverse changes in myocardial cell cytoplasm and mitochondria, leading to calcium overload, the opening of mitochondrial permeability transition pores, and disruption of mitochondrial homeostasis^26^,^27^. These changes further exacerbate acute inflammatory responses and excessive myocardial contraction, which collectively result in myocardial cell death. Clinically, extensive myocardial cell death manifests as acute myocardial infarction (AMI)^28^. The most common symptom is chest pain or discomfort, with patients often experiencing rest angina lasting more than 20 minutes, new-onset angina, reduced physical activity tolerance, or an increased frequency and duration of angina compared to prior episodes. However, animal studies indicate that in addition to early ischemia and hypoxia contributing to myocardial infarction, up to 50% of the infarct size in AMI patients may be attributable to reperfusion injury. Thus, beyond timely reperfusion therapy, mitigating or preventing reperfusion injury is a crucial aspect of managing myocardial infarction.

## 3. Mitochondria in cardiac cells

### 3.1. Mitochondrial DNA

The endosymbiotic hypothesis postulates that mitochondria evolved from free-living bacteria^38^. Primitive anaerobic eukaryotic cells engulfed these primitive mitochondria, establishing a mutually beneficial interaction. The discovery of mitochondrial DNA (mtDNA) and an independent mitochondrial translation system in the 1960s supports this theory^29^. Mitochondria possess their own distinct genetic code, which is circular in structure, serving as a molecular reminiscence of their bacterial ancestors. The vast majority of mitochondrial proteins (>99%) are produced by the host cell's nuclear DNA and are actively transported into mitochondria by mitochondrial membrane transport proteins^30^. The production of ATP, the cellular energy currency, occurs through the respiratory chain in mitochondria. It is worth noting that mtDNA mutations are associated with numerous hereditary familial cardiomyopathies in both adults and children.

### 3.2. Structure of mitochondrial membrane

Mitochondria, commonly referred to as the "powerhouse" of the cell, have long been recognized as the primary site for oxidative phosphorylation (OXPHOS), which generates the energy needed for cellular metabolism. Mitochondria are comprised of two membranes, namely the outer membrane (OMM) and the inner membrane (IMM). The IMM is further divided into the inner boundary membrane (IBM), which is in contact with the OMM and forms cristae. Cristae are connected to the IBM through narrow connections and extend into the mitochondrial matrix, thus increasing the surface area of the IMM. While the OMM faces the cytoplasm, the IMM extends into the mitochondrial matrix, which contains mtDNA^30^. The space between the inner and OMM, known as the intermembrane space (IMS), is also referred to as the intercompartment.

### 3.3. Distribution of mitochondria

The heart is a highly oxygen and ATP demanding organ that continuously synthesizes ATP to maintain cardiac function. The adult heart derives a significant amount of ATP for contraction by metabolizing various fuels such as fatty acids, glucose, lactate, ketones, pyruvate, and amino acids from two primary sources: mitochondrial OXPHOS and glycolysis. Generally, mitochondrial OXPHOS accounts for about 95% of the cardiac ATP demand, while glycolysis provides the remaining 5%^31^. Altered cardiac energy metabolism can affect heart failure severity, and varying degrees of mitochondrial dysfunction are commonly observed in failing hearts^32^,^33^.

To support the heart's high energy requirements, closely interconnected mitochondrial networks form within myocardial cells, comprising approximately 30% of the cell volume. Cardiac myocytes host two distinct subpopulations of mitochondria with different morphology and function: subsarcolemmal mitochondria (SSM), located beneath the sarcolemma^33^, and interfibrillar mitochondria (IFM), situated between the myofibrils. SSM and IFM exhibit contrasting morphological patterns in their cristae structures^33^. SSM has densely stacked lamellar cristae, constituting the majority (77%) and appearing wide and flat. In contrast, IFM align longitudinally along the myofibrils, occupying the space between the Z-lines and forming a network restricted by intermyofibrillar material. The cristae in IFM have a more variable shape, with some mitochondria (55%) possessing solely tubular cristae and others (24%) exhibiting a mixture of tubular and lamellar cristae^33^. Due to their specific subcellular localization, SSM and IFM perform distinct functions. IFM is responsible for providing ATP for contraction, while SSM primarily facilitates the active transport of electrolytes and metabolites through the sarcolemma to support ATP supply. Compared to SSM, IFM exhibits a higher respiration rate, enabling a 1.5-fold faster oxidation of all lipid and non-lipid substrates^33^. However, in terms of protein synthesis rates, the IFM subpopulation demonstrates lower synthesis rates compared to SSM. Moreover, specific ceramide distributions vary between SSM and IFM. The spatial positioning of mitochondria within cardiac myocytes may be associated with specific responses required for various physiological or pathophysiological stimuli, and differences between these two types of mitochondria have been validated in response to factors such as hypoxia, myocardial infarction, aging, diabetes, and metabolic hyperactivity-induced cardiac injury^33^-^35^.

## 4. Mitochondria in Hypoxic myocardial injury

### 4.1. Changes of mitochondria in hypoxic myocardial injury

When myocardial ischemia occurs, it can result in mitochondrial damage, leading to a dysfunction in OXPHOS. This dysfunction causes a severe energy crisis by reducing ATP production and damaging myocardial cell function. Additionally, calcium overload in mitochondria promotes the opening of Ca^2+^-dependent ion channels, which are widely distributed and mainly regulate the concentration of calcium ions inside and outside the cells. This affects the permeability of mitochondrial membranes and alters the membrane structure. As a result, apoptotic protein factors are released into the cytoplasm, causing damage to myocardial cells. Ultimately, this process triggers myocardial cell apoptosis and necrosis^32^,^36^.

Mitophagy, a bidirectional process, plays a crucial role in maintaining the normal physiological function of cells within normal ranges. However, excessive or insufficient mitophagy can contribute to the occurrence of related diseases. The process of mitophagy is tightly controlled and highly selective, regulated by proteins involved in mitochondrial fission and fusion, BCL-2 family proteins, and the PINK1/Parkin pathway^32^,^36^.

#### 4.1.1. Protective role of mitochondrial autophagy in hypoxic myocardial injury

Cardiac injury following AMI is a consequence of ischemia and reperfusion in specific regions of the myocardial tissue, ischemia and hypoxia lead to myocardial cell damage^37^. Mitophagy, which refers to the selective autophagy of mitochondria, serves as a quality control mechanism for damaged mitochondria and is a response to mitochondrial damage. It is believed that during hypoxic myocardial injury, mitophagy plays a protective role by removing damaged mitochondria and reducing their harmful effects on myocardial cells, including oxidative stress^11^,^38^. Mild mitochondrial oxidative stress occurs during ischemia itself. However, upon initiation of hypoxic and in the following hours and days, there is a significant generation of reactive oxygen species (ROS), leading to oxidative stress, exacerbating mitochondrial damage^39^. The damaged mitochondria further stimulate the production and accumulation of ROS. In the meantime, defective mitophagy fails to eliminate the damaged mitochondria caused by I/R injury, resulting in mitochondrial genomic instability, inhibition of the electron transport chain (ETC), impaired mitochondrial biogenesis, oxidation of cardiac phospholipids, oxidative stress, opening of the mitochondrial permeability transition pore (mPTP), and ultimately, mitochondrial apoptosis, directly triggering cell death^40^-^42^.

Evidence suggests that mitochondrial fission is capable of meeting high energy demands. It involves the division of mitochondria into two daughter mitochondria and facilitates the removal of damaged mitochondria from myocardial cells, thereby preserving the segregation of damaged mitochondria from the entire cellular network. On the other hand, mitochondrial fusion primarily involves the merging of healthy mitochondrial components to repair and regenerate mitochondria^43^. As a result, complete separation through fission and repair through fusion may enhance the proper elimination of damaged mitochondria through mitochondrial autophagy and the renewal of healthy organelles. Conversely, interrupting the fission process may promote the fusion of healthy and damaged mitochondrial components, leading to an acceleration of mitochondrial dysfunction within myocardial cells^44^,^45^. Mounting evidence suggests a reciprocal dependence between mitochondrial fission and mitochondrial autophagy, which is a specific form of autophagy that targets mitochondria, particularly with regard to Drp1^41^. Decreasing the expression of Drp1 not only prompts mitochondrial fusion but also results in an accumulation of damaged mitochondria due to the inhibition of mitochondrial autophagy, thereby exacerbating myocardial infarction^46^.

#### 4.1.2. Negative effects of mitochondrial autophagy in hypoxic myocardial injury

Complete mitochondrial autophagy plays a critical role in regulating hypoxic myocardial injury. It contributes to the regulation of mitochondrial oxidative stress, signal transduction within the mitochondrial network, endothelial protection, and mitochondria-related cell death. Maintaining an appropriate level of mitochondrial autophagy and timely clearance of damaged mitochondria are essential to prevent excessive accumulation of reactive oxygen species (ROS) and protect the integrity of myocardial cells^11^. Autophagy is generally considered a protective response that maintains cellular homeostasis by eliminating damaged proteins and aged organelles^37^. However, during hypoxic myocardial injury, autophagy may not always provide a protective effect. Excessive mitochondrial autophagy can result in the excessive removal of mitochondria, impairing cellular function and exacerbating myocardial cell damage. When mitochondrial autophagy becomes overactivated and uncontrolled, it leads to the accumulation of autophagosomes, abnormal degradation of key proteins and organelles, a decrease in the number of mitochondria in myocardial cells, and a disruption in energy supply to the cells^47^,^48^.

### 4.2. Pathways related to mitochondrial autophagy

#### 4.2.1. PINK1-Parkin-mediated mitochondrial autophagy

PINK1 and Parkin are involved in the selective removal of damaged mitochondria through the induction of mitochondrial autophagy^49^. Normally, PINK1 is imported into healthy mitochondria with normal membrane potential and degraded through protein hydrolysis, leading to its low levels in cardiac cells^50^. However, during stimuli such as ischemia and hypoxia, the mitochondrial metabolism undergoes changes that cause a depolarization of the mitochondrial membrane potential and inhibit the normal functioning of relevant mitochondrial hydrolases. Consequently, PINK1 degradation is blocked, resulting in its accumulation in the outer mitochondrial membrane (OMM). This accumulation triggers the ubiquitination and activation of Parkin. Subsequently, Parkin ubiquitinates mitochondrial proteins such as Mfn1, Mfn2, and others. The ubiquitinated proteins are then recognized by autophagy receptor proteins, including OPTN, p62, and NDP52, leading to the sequestration of damaged mitochondria into autophagosomes. These autophagosomes subsequently fuse with lysosomes for degradation^51^.

#### 4.2.2. FUNDC1-mediated mitochondrial autophagy

FUNDC1, a receptor for mitochondrial autophagy, is situated at the interface between the outer mitochondrial membrane (OMM) and the ER-mitochondria associated membranes (MAM)^52^. It possesses an LC3 interacting region (LIR) motif, which enables the binding to LC3. This binding facilitates the expansion of mitochondrial autophagosomes and the elimination of impaired mitochondria. Under normal circumstances, FUNDC1 undergoes phosphorylation at position 13 by casein kinase 2 (CK2) and at position 18 by src family kinase (Src). Phosphorylated FUNDC1 exhibits reduced interaction with LC3^53^. In hypoxic conditions, FUNDC1 not only facilitates the formation of MAMs and excessive mitochondrial Ca^2+^ accumulation, resulting in mitochondrial autophagy, but also promotes mitochondrial division by recruiting DRP1. This recruitment promotes mitochondrial fission and the occurrence of mitochondrial autophagy^40^,^54^.

#### 4.2.3. BNIP3/NIX-mediated mitochondrial autophagy

BNIP3 and BNIP3L/NIX are mitochondrial proteins that belong to the BCL2 (B-cell CLL/lymphoma)-related family. They function as receptors for autophagy, directly binding to LC3 on the autophagosome, thereby initiating mitochondrial autophagy. The process of BNIP3/NIX-mediated mitochondrial autophagy primarily occurs during the early stages of ischemic heart disease and is characterized by excessive production of reactive oxygen species (ROS)^55^. In conditions of hypoxia, myocardial hypertrophy, or ischemia, the expression of BNIP3L increases, leading to the induction of mitochondrial autophagy or cell apoptosis^56^. In an I/R mouse model, the absence of BNIP3 restricts post-myocardial infarction ventricular remodeling by decreasing apoptosis in the infarct border zone. Additionally, NIX also plays a role in heart disease, but unlike BNIP3, its main function is associated with the induction of cardiac hypertrophy^57^.

## 5. Relationship between mitochondrial unfolded protein response and hypoxic myocardial injury

### 5.1. Mitochondrial unfolded protein response

Mitochondria serve as the cell's powerhouse, playing a crucial role in maintaining the normal function of cardiomyocytes. The functionality of mitochondria depends on the integrity of their proteome. Ischemia and hypoxia in cardiomyocytes lead to mitochondrial dysfunction, further impairing the ability of mitochondria to fold proteins properly. Consequently, a large amount of misfolded and unfolded proteins accumulate within the mitochondria, disrupting proteostasis^58^. In response, cells activate the mitochondrial unfolded protein response (mtUPR) as a protective mechanism^59^. The mtUPR facilitates communication between the mitochondria and the nucleus, restoring proteostasis by reducing the protein-folding burden and eliminating toxic proteins. This process enhances cell survival and, to some extent, aids in the repair of damaged mitochondria^60^.

### 5.2. ATF5/ATF4/CHOP and mtUPR

Activating transcription factor 5 (ATF5) provides the first *in vivo* evidence of its role in the mammalian UPRmt and is also the first confirmation that UPRmt serves as a target for cardioprotective drugs^61^. ATF5, the mammalian homolog of ATFS-1, specifically induces hsp-60 and activates mtUPR in mammalian cells. Although ER stress can activate ATF5, studies show that ATF5 is the only mitochondria-specific transcription factor activated during mitochondrial stress, and it does not induce chaperones during ER stress^62^. Consequently, ATF5 is considered a key regulator of UPRmt. ATF5 belongs to the bZip family of transcription factors, containing a basic DNA-binding region and a leucine zipper structure for dimerization. It can homodimerize with other ATF5 proteins or heterodimerize with C/EBPβ, binding specific DNA sequences to regulate target gene expression^63^. ATF5 localizes to the mitochondria and supports mitochondrial survival. During mitochondrial stress, ATF5 translocates to the nucleus using its mitochondrial targeting sequence (MTS), nuclear export sequence (NES), and nuclear localization sequence (NLS), where it activates nuclear transcriptional responses^64^.

In addition to ATF5, ATF4 and CCAAT/enhancer-binding protein homologous protein (CHOP) also serve as important transcription factors during mitochondrial stress. ATF4 is phosphorylated and binds to the promoters of mtUPR genes to activate mtUPR. Additionally, ATF4 contributes to the induction of CHOP expression^65^. CHOP, in turn, dimerizes with C/EBPβ and binds to the promoter regions of mtUPR genes, acting as a key regulator of the mitochondrial stress response^66^. ATF4 and CHOP work together with ATF5 to activate UPRmt^67^.

The ATF5/ATF4/CHOP pathway is the canonical signaling mechanism of mtUPR and maintains mitochondrial protein stability by regulating the expression of mitochondrial chaperones and proteases^68^. Chaperones stabilize unfolded or partially folded polypeptides, preventing their aggregation and assisting in proper protein folding, thereby protecting cells from damage. Proteases, on the other hand, maintain mitochondrial homeostasis by degrading damaged or misfolded proteins. The degraded peptides are subsequently transported from the mitochondrial matrix to the intermembrane space via HAF-1, a mitochondrial inner membrane ABC transporter, before diffusing into the cytoplasm^69^.

The HSP family is one of the earliest and most extensively studied molecular chaperone families^70^. HSP60, located in mitochondria, helps maintain the mitochondrial respiratory chain. Its monomers arrange into heptameric rings, which, together with co-chaperone HSP10 and ATP, form a complex that encapsulates misfolded or unfolded proteins. This complex facilitates the proper folding of proteins, thereby maintaining proteostasis^71^. Mitochondrial heat shock protein 70 (mtHSP70) resides mainly in the mitochondria, containing a nucleotide-binding domain and a substrate-binding domain, and is involved in refolding unfolded proteins^72^. Under hypoxic conditions, mtHSP70 can also bind to specific transcription factors like HIF-1α, aiding its localization to the mitochondrial outer membrane. HIF-1α then interacts with voltage-dependent anion-selective channel 1 (VDAC1) and hexokinase-II (HK-II) to prevent apoptosis and promote survival^73^. Additionally, studies have revealed that molecular chaperones, such as HSP22 and HSP60 or HSP70 and TRAP1, interact with each other to co-regulate mtUPR^74^.

Proteases are also crucial signaling molecules for UPRmt. Over 40 proteases have been identified in various mitochondrial compartments, with ClpP and LONP1 being the most critical ATP-dependent proteases in the eukaryotic mitochondrial matrix^75^,^76^. LONP1 (Lon Peptidase 1) is composed of a hexameric cylindrical structure and a large degradation chamber responsible for breaking down misfolded proteins or aggregates into short peptides, thus preventing their accumulation in the mitochondria and preserving proteostasis^77^. However, LONP1 does not degrade folding intermediates of mitochondrial proteins, suggesting that mitochondria prioritize the chaperone system to promote proper protein folding. Only when protein folding fails and cannot be corrected by chaperones does LONP1 act as the "last resort" for protein degradation^78^. ClpP, structurally similar to LONP1, is a tetradecameric cylindrical protease that selectively degrades misfolded or damaged proteins by binding to adapter proteins like ClpX. ClpX is an essential cofactor that helps recognize protein substrates and deliver them to ClpP for degradation^79^.

### 5.3. Sirt3-FOXO3a-SOD2 pathway

Silent information regulator factor 2-related enzyme 3 (Sirt3) is a mitochondrial nicotinamide adenine dinucleotide (NAD)-dependent histone deacetylase, capable of deacetylating both histone and non-histone targets. SIRT3 is localized to the mitochondrial matrix, and its expression is upregulated in response to the accumulation of unfolded or misfolded proteins. SIRT3 promotes the deacetylation of the transcription factor forkhead box protein O3a (FOXO3a), enhancing FOXO3's nuclear translocation and transcriptional activity. FOXO3 subsequently regulates the expression of manganese superoxide dismutase (SOD2) and catalase, leading to antioxidant effects that reduce mitochondrial superoxide levels. This reduction in superoxide helps to lower oxidative stress and minimize protein oxidation and misfolding^80^,^81^.

### 5.4. Estrogen receptor alpha (ERα) -NRF1-HTRA2 and UPRmt

Unfolded or misfolded proteins can accumulate in both the mitochondrial matrix and the intermembrane space (IMS), activating the matrix unfolded protein response (mtUPR) and the IMS-specific unfolded protein response, respectively^82^,^83^. The IMS contains fewer heat shock proteins and proteases compared to the matrix, making it more susceptible to stress. Under IMS stress, protein kinase B (PKB/AKT) phosphorylates and activates estrogen receptor α (ERα), which further regulates the expression of nuclear respiratory factor 1 (NRF1) and the IMS protease high-temperature requirement A2 (HTRA2, also known as OMI). NRF1 binds to SIRT7, transporting it to the nucleus where it modifies chromatin, while HTRA2 degrades misfolded proteins. The mtUPR in the IMS is highly specific, as both HTRA2 and the 26S proteasome are selectively activated to degrade damaged or misfolded proteins. Unlike the matrix mtUPR, the IMS mtUPR does not induce CHOP or HSP60 in this ERα-dependent process^84^. Additionally, due to gender differences in ERα expression, the activation of the IMS mtUPR pathway exhibits a sex-specific effect, with the ERα-NRF1-HTRA2 axis providing stronger mitochondrial protection in females than in males^85^.

There is a synergistic interaction between different mtUPR pathways, which collectively work to clear misfolded proteins and maintain proteostasis. Research shows that when ERα is inhibited, the CHOP axis of the mtUPR can compensate, helping to preserve mitochondrial integrity.

### 5.5. Mitochondrial unfolded protein response and hypoxic myocardial injury

**Figure 4:**
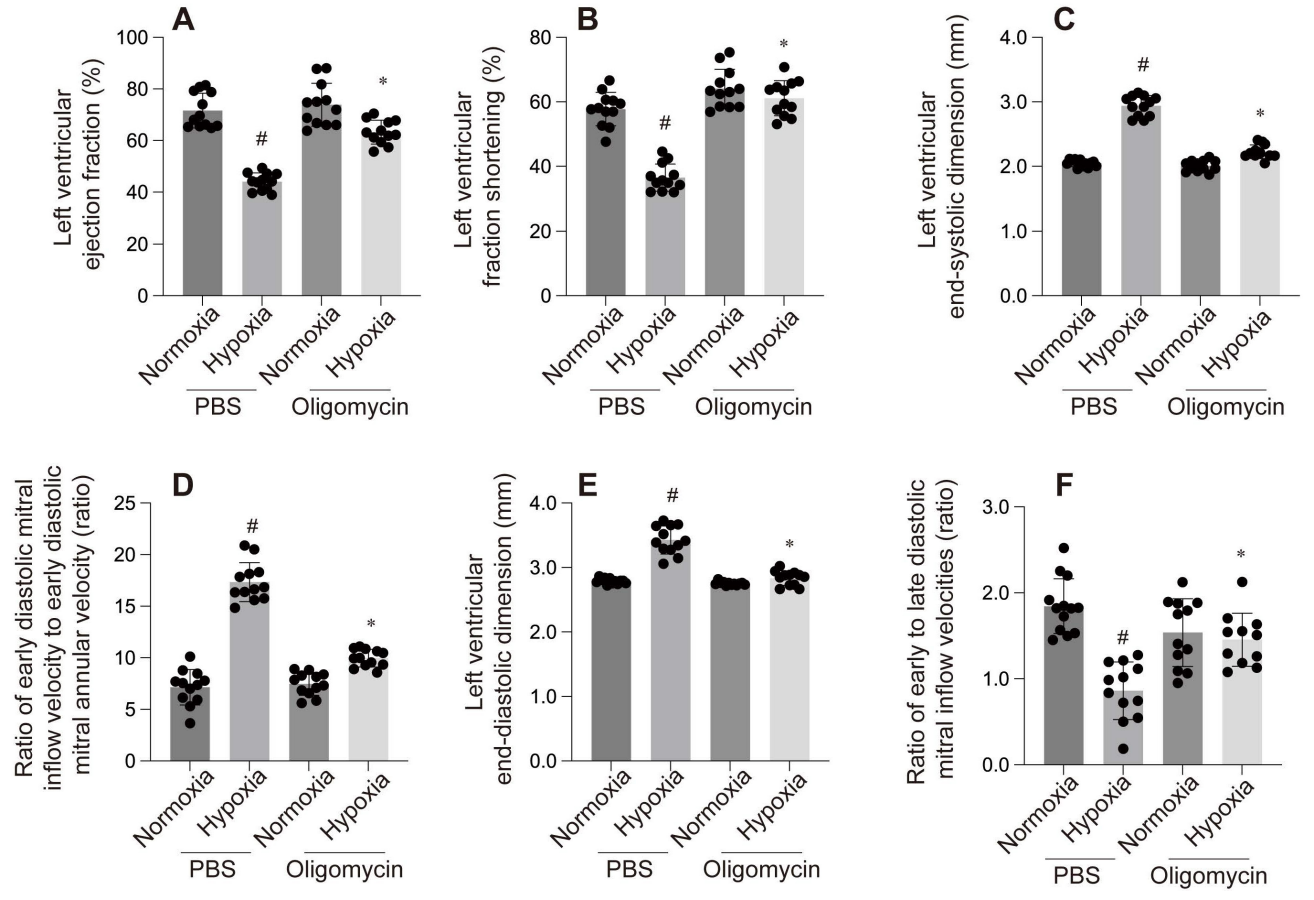
A-F: These panels compare the effect of PBS and Oligomycin under normoxia and hypoxia on various cardiac parameters: #p < 0.05 vs PBS+Normoxia group, *p<0.05 vs Olgomycin+Normoxia group.

**Figure 5:**
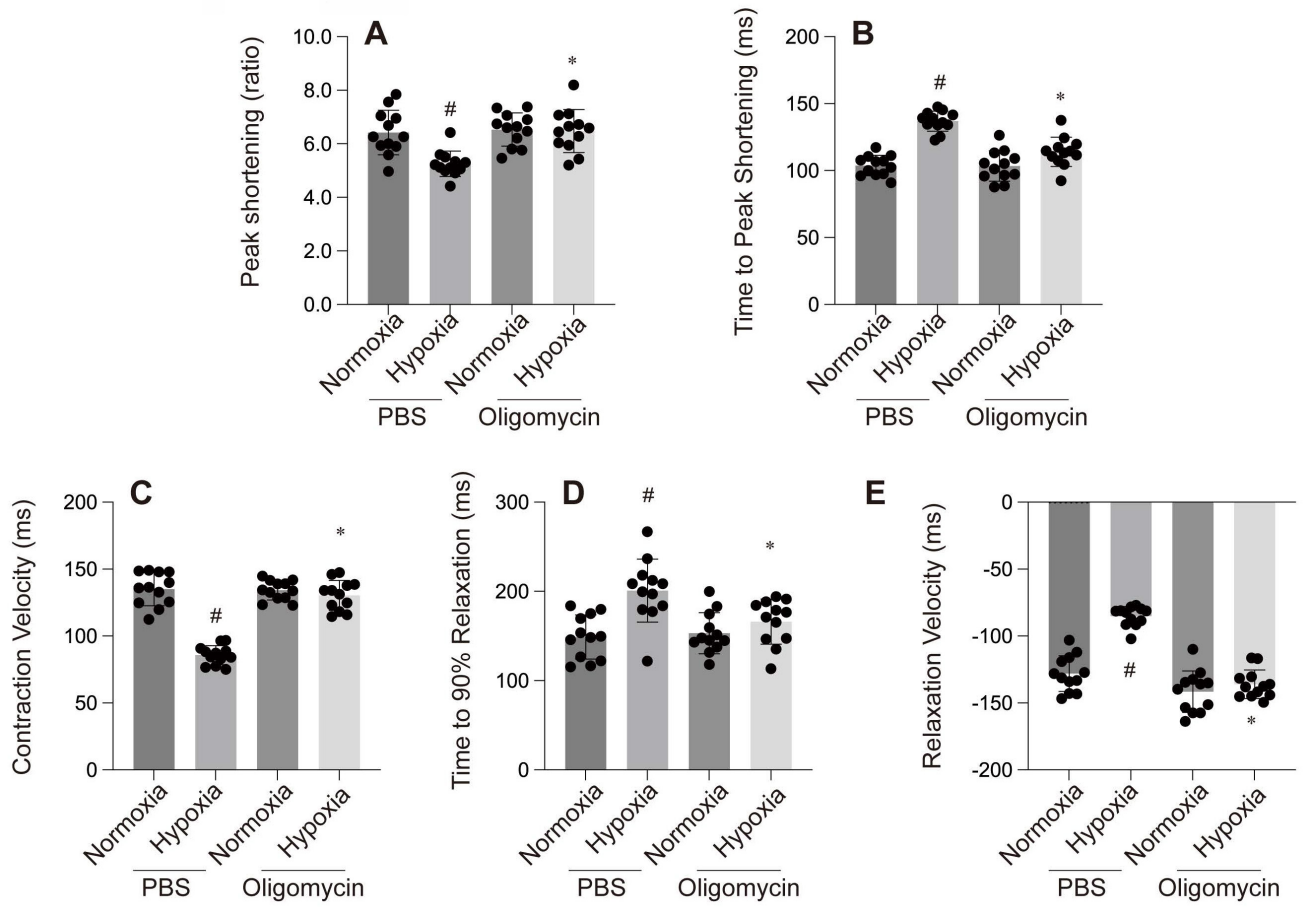
A-E: Contractility and relaxation parameters. #p < 0.05 vs PBS+Normoxia group, *p<0.05 vs Olgomycin+Normoxia group.

**Figure 6:**
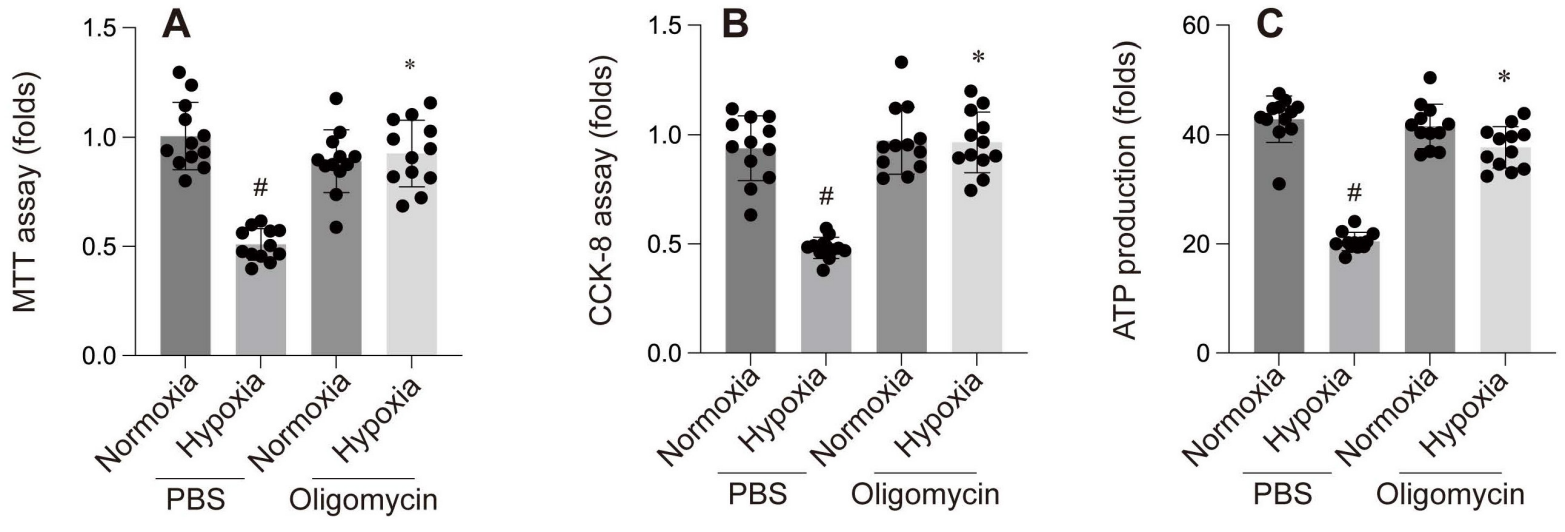
A-C: Cellular assays: A & B: MTT and CCK-8 assays show reduced cell viability under hypoxia with PBS, while Oligomycin enhances cell survival. C: ATP production is reduced under hypoxia with PBS but is partially restored by Oligomycin. #p < 0.05 vs PBS+Normoxia group, *p<0.05 vs Olgomycin+Normoxia group.

Mitochondria are the primary oxygen consumers within cells, and they are the first organelles to be affected during hypoxia. The decline in oxygen levels leads to the failure of cytochrome C oxidase, resulting in reduced ATP levels. In addition to generating reactive oxygen species (ROS), hypoxia disrupts all ATP-dependent cellular processes, such as protein transport, synthesis, folding, and the maintenance of the electrochemical gradient, which in turn induces cardiomyocyte damage and further impairs cardiac function. In one of our studies, hypoxia was observed to cause left ventricular dilation, dysfunction in both systolic and diastolic performance, and a reduced ejection fraction (Figure 4-6).

Research has shown that hypoxia-induced cellular damage is closely associated with the disruption of mitochondrial proteostasis^86^. In C. elegans, hypoxia disrupts mitochondrial proteostasis by impairing ATP production and inhibiting the translation of nuclear-encoded proteins, leading to misfolded proteins in the mitochondria. The activation or suppression of mtUPR under hypoxic conditions is an ATP-dependent process, which varies depending on the specific cellular context and experimental model. Yan *et al.* screened 161 hypoxia-related RNAi genes in C. elegans and found that only five RNAi knockdowns significantly activated mtUPR under hypoxia^86^. In contrast, Mao *et al.* discovered that paclitaxel significantly reduced the basal expression of the mitoUPR reporter gene, thereby inducing axonal injury. In our own study, we found that hypoxia reduced the expression of mtUPR-related genes (CHOP, LONP1, mtDNAj, ATF5) in cardiomyocytes^87^.

The mitochondrial unfolded protein response (mtUPR) is critical for maintaining mitochondrial homeostasis. An *et al.* identified mtUPR as a potential therapeutic target for ischemic stroke, showing that oligomycin-induced mtUPR protects neurons from oxygen-glucose deprivation, thereby reducing infarct size and improving neurological outcomes. When mtUPR was blocked, the corresponding neuroprotective effects disappeared^88^. In our study, we also observed that, compared to the control group, the oligomycin group exhibited reduced left ventricular dilation under hypoxic conditions, alleviated cardiac systolic and diastolic dysfunction, and increased cell viability and ATP production. These findings suggest that oligomycin exerts protective effects on cardiomyocytes under hypoxia. This protective mechanism may be related to the mtUPR's ability to modulate nuclear gene transcription, reduce the burden of unfolded proteins within mitochondria, clear misfolded abnormal proteins, and mitigate ischemic-hypoxic injury to cardiomyocytes by facilitating the aggregation of misfolded proteins. However, further investigation is required to fully elucidate these processes.

## 7. Drugs targeting mitochondria to treat myocardial damage caused by hypoxia

### 7.1. Quercetin

Quercetin, a flavonol derived from plants, belongs to the polyphenol and flavonoid classes of compounds and is widely found in various fruits, vegetables, and grains. Numerous studies have demonstrated that quercetin and its major derivatives, such as rutin, isoquercetin, and hyperoside, provide various health benefits to the human body^89^. Specifically, their cardioprotective effects have been consistently confirmed in both cellular and animal models^90^,^91^.

*In vitro* experiments have shown that quercetin improves oxidative damage in rat cardiomyocytes by reducing reactive oxygen species (ROS) production, decreasing the levels of stress-activated protein kinase (SAPK)/c-Jun N-terminal kinase (JNK), heat shock protein 27 (Hsp27), and caspase-3, while enhancing cardiomyocyte viability^90^. Moreover, pre-treating primary neonatal rat cardiomyocytes with quercetin significantly increased cell survival in a hypoxia/reoxygenation model. This was attributed to reduced ROS production, prevention of mitochondrial membrane potential collapse, inhibition of mitochondrial permeability transition pore (mPTP) opening, and mitigation of subsequent apoptosis. Additionally, quercetin is rapidly metabolized *in vivo*^23^,^92^. Encapsulating quercetin in poly(lactic-co-glycolic acid) (PLGA) nanoparticles effectively maintains mitochondrial oxygen consumption rates and membrane potential, resulting in higher ATP production, preservation of mitochondrial function, and protection of cardiomyocytes^91^,^93^.

In addition to quercetin itself, its derivatives have also been found to exhibit cardioprotective effects^94^. Dihydroquercetin (DHQ), a quercetin derivative, significantly alleviates cardiac dysfunction caused by ischemia-reperfusion (I/R) injury in rats. DHQ scavenges free radicals, reduces lipid peroxidation, and enhances the activity of antioxidant enzymes. Western blot analysis showed that DHQ inhibits apoptotic pathways by suppressing the expression of pro-apoptotic proteins CHOP, caspase-12, and p-JNK. Moreover, DHQ alleviates endoplasmic reticulum stress by reducing the expression levels of GRP78, p-PERK, and p-eIF2α, while increasing HO-1 expression and promoting the interaction between Nrf2 and antioxidant response elements^94^. Another derivative, isoquercetin (isoQC), shows potential as a therapeutic agent for preventing cardiomyocyte apoptosis induced by I/R injury. In I/R-related models, isoQC enhanced the viability of H9C2 cells in a dose-dependent manner and inhibited apoptosis after I/R injury. In isoQC-treated H9C2 cells subjected to I/R injury, Bax expression was reduced, while Bcl-2 expression increased. Furthermore, isoQC protected cardiomyocytes from hypoxia/reoxygenation-induced injury via a mitochondrial-dependent pathway. In addition to reducing ROS, isoQC also prevented the release of cytochrome c following I/R injury and significantly decreased caspase-9 and caspase-3 levels in H9C2 cells after I/R injury^93^.

### 7.2. Erythropoietin (SS-31)

Elamipretide is an aromatic cationic, cell-permeable tetrapeptide that belongs to a new class of mitochondria-targeted drugs. It targets mitochondria by binding to cardiolipin, stabilizing the mitochondrial membrane and cytochrome c, thereby improving energy metabolism and reducing the production of reactive oxygen species (ROS). ROS can activate mitochondrial proteases and the mitochondrial unfolded protein response (mtUPR), triggering dynamic changes in mitochondria and accelerating the accumulation of oxidative byproducts. These byproducts, such as lipid peroxides and protein carbonyl compounds, cause oxidative damage to cellular membranes, proteins, and DNA, further impairing the structure and function of cardiomyocytes. By reducing ROS production, Elamipretide effectively decreases the formation of oxidative byproducts, preventing damage to cardiomyocytes during hypoxia or oxidative stress, thereby protecting mitochondrial function and maintaining cellular homeostasis^95^.

Additionally, Elamipretide has been used to treat ventricular failure and non-failing tissues in both children and adults. Studies have shown that in freshly transplanted heart tissues, treatment with Elamipretide significantly improves mitochondrial oxygen flux, the activity of complex I (CI) and complex IV (CIV), and in-gel activity of supercomplex assembly^96^. In an ischemia-reperfusion (IR) study, Elamipretide inhibited the levels of oxidative stress markers (NOX-1, NOX-2, and oxidized proteins) induced by menadione and significantly increased the expression of SIRT1 and SIRT3. By mitigating inflammation and oxidative stress and reducing mitochondrial protein oxidation and misfolding, Elamipretide preserved mitochondrial integrity, ultimately improving myocardial function^38^,^97^.

### 7.3. BRAWNIN

**Figure 7:**
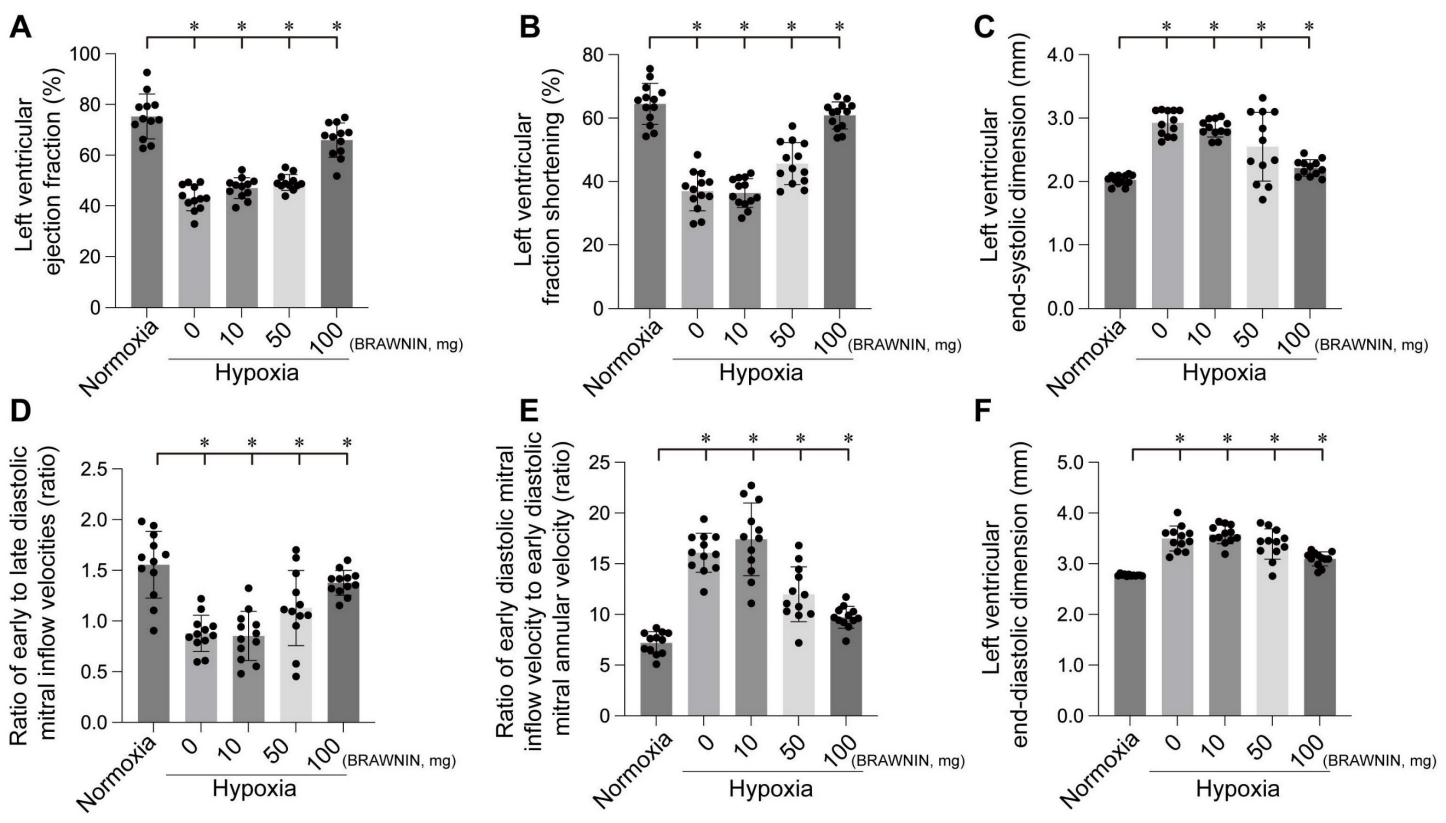
BRAWNIN intervention improves left ventricular functional and structural abnormalities induced by hypoxia. *p < 0.05.

**Figure 8:**
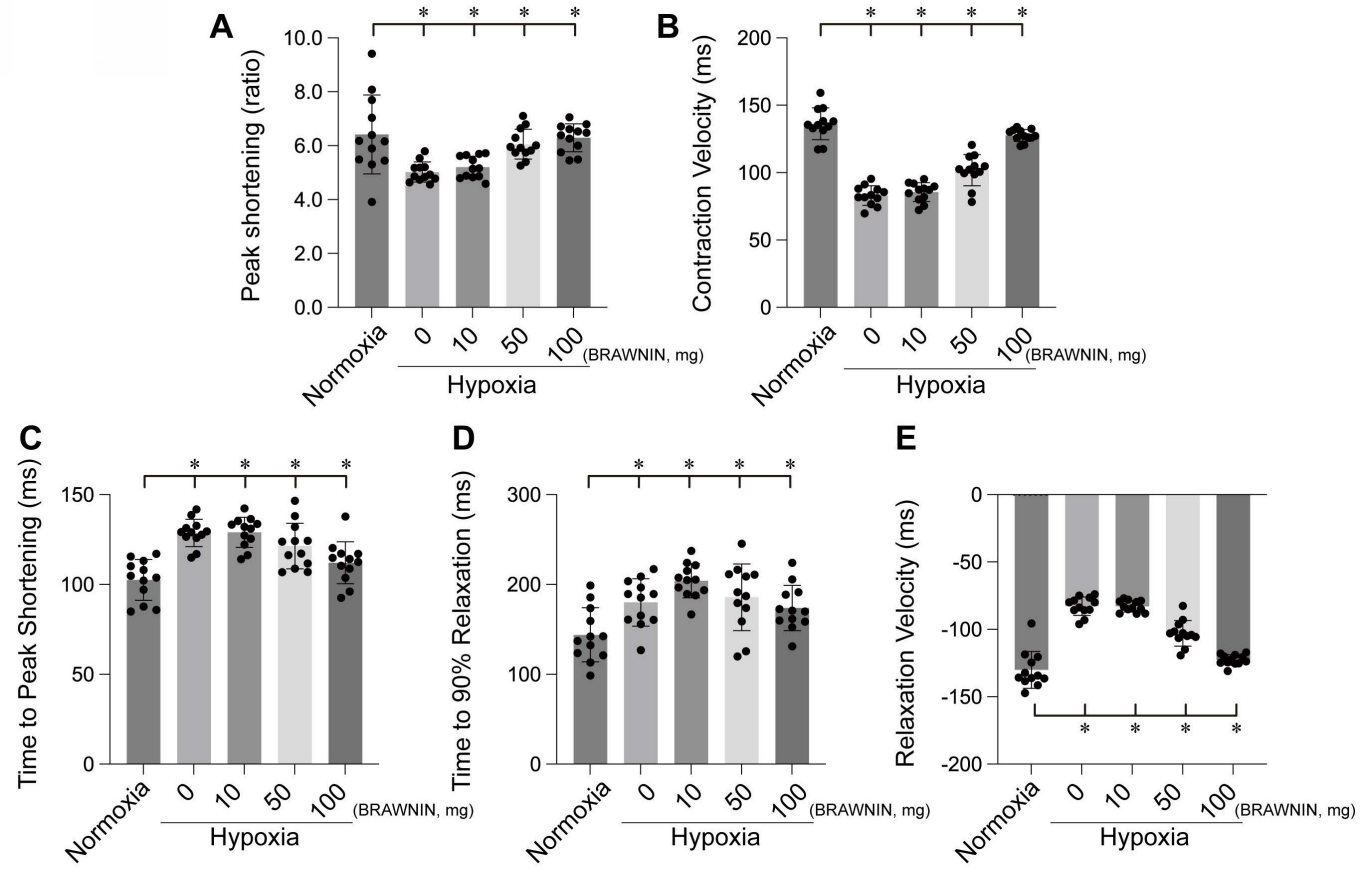
BRAWNIN alleviates hypoxia-induced myocardial contractile and diastolic dysfunction. *p < 0.05.

BRAWNIN (BR) is a small mitochondrial peptide consisting of 71 amino acids, encoded by the C12orf73 gene. Regulated by the AMPK signaling pathway, BR is involved in the assembly of respiratory complex III (CIII), thereby influencing mitochondrial oxidative phosphorylation (OXPHOS) and ATP synthesis.^97^ BR is highly expressed in tissues with elevated mitochondrial activity, such as skeletal muscle, heart, and brown adipose tissue, but is nearly absent in white adipose tissue^98^. BR is crucial for cellular bioenergetics, and its deficiency can lead to mitochondrial dysfunction, potentially causing conditions such as lactic acidosis, growth retardation, and metabolic disorders. Targeting BR as a novel therapeutic strategy holds great potential for treating diseases associated with mitochondrial dysfunction, such as diabetes, heart disease, and mitochondrial myopathies. Our experiments also confirmed that BRAWNIN effectively alleviates structural and functional abnormalities of the heart under hypoxic conditions (Figure 7-8) by modulating cardiac contractile and diastolic function^99^.
